# Chromogenic *in situ* hybridization reveals specific expression pattern of long non-coding RNA *DRAIC* in formalin-fixed paraffin-embedded specimen

**DOI:** 10.1016/j.ncrna.2023.11.004

**Published:** 2023-11-11

**Authors:** Kouhei Sakurai, Seiji Yamada, Rika Ito, Mako Ochiai, Tatsuya Ando, Yasuhiro Sakai, Taku Kato, Hiroyasu Ito

**Affiliations:** a Department of Joint Research Laboratory of Clinical Medicine, School of Medicine, Fujita Health University, Toyoake, Aichi, 470-1192, Japan; b Department of Diagnostic Pathology, School of Medicine, Fujita Health University, Toyoake, Aichi, 470-1192, Japan; c Faculty of Medical Technology, School of Medical Sciences, Fujita Health University, Toyoake, Aichi, 470-1192, Japan

**Keywords:** Long non-coding RNA, *DRAIC*, Chromogenic *in situ* hybridization, RNAscope, Tissue microarray, Neuroendocrine differentiation

## Abstract

Long non-coding RNA (lncRNA) plays an important role in the regulation of gene expression in normal and cancer cells. We previously discovered a novel tumor-suppressive lncRNA, *DRAIC*, in prostate cancer cells. Subsequent studies have demonstrated that *DRAIC* is dysregulated in various malignancies and exhibits a tumor-suppressive or pro-oncogenic function. However, details regarding its expression pattern in normal and cancerous tissues remain largely unknown. In this study, we performed chromogenic *in situ* hybridization (CISH) using RNAscope technology to assess *DRAIC* expression in formalin-fixed paraffin-embedded (FFPE) specimens. In the neuroendocrine-differentiated cancer cell line VMRC-LCD, CISH revealed a diffuse localization of *DRAIC* in the cytoplasm as well as specific accumulation in the nuclear compartment. *DRAIC* expression was comprehensively analyzed using tissue microarrays containing 89 normal and 155 tumor tissue samples. *DRAIC* was weakly expressed in normal epithelial cells of the colon, bronchiole, kidney, prostate, and testis. Conversely, *DRAIC* was moderately to highly expressed in some cancer tissues, including prostate adenocarcinoma, invasive ductal carcinoma of the breast, neuroendocrine carcinoma of the esophagus, lung adenocarcinoma, and small cell lung carcinoma. While *DRAIC* knockdown did not affect VMRC-LCD cellular viability and invasive ability, gene expression related to the neuroendocrine and cancer-related pathways was altered. Our expression analysis revealed the specific expression pattern of *DRAIC* in normal and cancerous FFPE tissues. The results presented here may lead to the elucidation of additional novel functions of *DRAIC*.

## 1 Introduction

Recent advances in transcriptomic technology have revealed that the vast majority of the human genome is transcribed to produce non-coding RNA, which is not translated into proteins [1]. Along with well-characterized conventional non-coding RNAs such as tRNA and rRNA, various other types of non-coding RNAs are intracellularly expressed. Among these, long non-coding RNAs (lncRNAs) are defined as RNAs longer than 200 nucleotides in length with no open reading frame. While the total number of lncRNA genes remains unclear, it has been estimated that the number exceeds 100,000 in a human cell [2]. Although a few lncRNAs have been functionally characterized, some have been demonstrated to play biological roles in physiological processes, including development, cellular differentiation, and homeostasis [3]. Therefore, the abnormal expression of a specific lncRNA is closely linked to the initiation and progression of diseases such as cancer.

Each lncRNA is expressed in the nucleus, cytoplasm, or both and can interact with other molecules, such as nucleic acids and proteins, to perform its functions. For example, nuclear lncRNAs (e.g., *NEAT1* and *MALAT1*) are involved in chromatin remodeling, gene transcription, and RNA splicing through their interaction with transcription factors and RNA-binding proteins [4,5]. Cytoplasmic lncRNAs (e.g., *TINCR* and LINC00152) regulate the mRNA and protein turnover, modulate molecular signaling activity, and sequester microRNAs (miRNAs) [6,7]. Because the functions of lncRNAs are dependent on their subcellular localization and binding partners, it is essential to identify their distribution inside a cell to determine their biological roles.

Previously, we identified a novel tumor-suppressive lncRNA, *DRAIC* (Downregulated RNA in cancer, inhibition of cell invasion and migration), which is a 1.7 kb spliced and polyadenylated RNA that was predominantly expressed in the cytoplasm of prostate cancer cell lines [8]. We demonstrated that *DRAIC* had tumor-suppressive effects and its high expression was a good prognostic indicator in patients with prostate adenocarcinoma [8]. Subsequently, *DRAIC* was revealed to interact with subunits of the IκB kinase (IKK) complex to inhibit NF-κB activity and suppress prostate cancer progression [9]. Furthermore, the tumor-suppressive mechanisms of *DRAIC* have been reported in glioblastoma [10], gastric adenocarcinoma [11], retinoblastoma [12], and triple-negative/basal subtype of breast carcinoma [13]. Conversely, pro-oncogenic functions of *DRAIC* have been demonstrated in nasopharyngeal carcinoma [14] and lung adenocarcinoma [15]. Although the molecular mechanisms of the contradictory roles of *DRAIC* have not been fully elucidated, they might be attributed to its versatility in regulating multiple pathways that include autophagy [10,16], protein translation [10], ubiquitin modification [11], and immune cell infiltration [17]. To understand its complex mode of action [18], it is important to analyze the expression level and subcellular localization of *DRAIC* in each cell line and tissue.

Archived formalin-fixed paraffin-embedded (FFPE) samples are invaluable resources for identifying pathological biomarkers. Chromogenic *in situ* hybridization (CISH) is an analysis method to visually detect RNA in FFPE tissues at the single-cell level. We have demonstrated the efficacy of CISH to analyze the miRNA expression patterns in normal and cancerous FFPE tissue samples [19,20]. Although various *in situ* hybridization methods for lncRNAs have been developed [21], detecting lncRNAs in FFPE specimens remains challenging because the longer transcript is easily degraded during formalin fixation [22]. Furthermore, CISH technology with increased sensitivity is required to visualize lncRNAs because their expression level is typically lower than that of mRNAs [23]. To overcome these limitations, we performed CISH utilizing RNAscope technology [24] to detect *DRAIC* in FFPE cell lines and tissue microarrays (TMAs). The RNAscope CISH successfully visualized *DRAIC* expression, thus providing novel insights into the role of *DRAIC* in a subset of malignancies.

## 2 Material and methods

### 2.1 Cell culture

The VMRC-LCD, 22Rv1, and PC3 cell lines were obtained from the Japanese Collection of Research Bioresources Cell Bank (Osaka, Japan), the European Collection of Authenticated Cell Cultures (Salisbury, UK), and the American Type Culture Collection (Manassas, VA, USA), respectively. VMRC-LCD cells were maintained in Dulbecco's Modified Eagle Medium (DMEM) (Thermo Fisher Scientific, Waltham, MA, USA) containing 10 % fetal calf serum (FCS) (Thermo Fisher Scientific), and penicillin/streptomycin (P/S) (FUJIFILM Wako Chemical Corporation, Osaka, Japan), while the 22Rv1 and PC3 cells were maintained in RPMI-1640 (Thermo Fisher Scientific) containing 10 % FCS and P/S.

For the pathological analysis, the cells were washed twice using phosphate-buffered saline (PBS) (Thermo Fisher Scientific) without trypsin treatment and fixed using 10 % neutral buffered formalin (FUJIFILM Wako Chemical Corporation) for 3 h at room temperature (RT). The fixed cells were embedded in paraffin after being gelled with 1 % sodium alginate and 1 M CaCl_2_, as previously reported [25]. The 3-μm thick sections were mounted on silane-coated slides (New silane 3) (MUTO PURE CHEMICAL CO., L.T.D., Hongo, Tokyo, Japan).

### 2.2 Transfection

VMRC-LCD cells at 3 × 10^5^ cells/well were seeded in a 6-well plate. At 24 h after seeding, transfection with siRNA (20 nmol/L) was performed using Lipofectamine RNAiMax transfection reagent (Thermo Fisher Scientific). The negative control siRNA (si-NC: #51-01-14-03) and two siRNAs against *DRAIC* (si-*DRAIC*1: hs. Ri.LOC145837.13.1; si-*DRAIC*2: hs. Ri.LOC145837.13.2) were obtained from Integrated DNA Technologies (Coraville, IA, USA).

### 2.3 Cell viability assay

VMRC-LCD cells at 1 × 10^4^ cells/well were seeded in a 96-well plate. At 24 h after seeding, siRNA transfection was performed as described above. The cells were subjected to a cell viability assay at the designated time point using WST-8 (DOJINDO LABORATORIES, Kumamoto, Japan) according to the manufacturer's instructions. The 450-nm absorbance of samples was measured using Sunrise Rainbow (Tecan, Männedorf, Switzerland).

### 2.4 Matrigel invasion assay

VMRC-LCD cells at 1 × 10^5^ cells/well after transfecting siRNA were seeded into 24-well Matrigel Invasion Chamber (#354480, Corning, NY, USA) in serum-free DMEM. Ten percent FCS was added only to the lower compartment. After incubation for 48 h, the noninvaded cells were removed from the upper surface of the membrane by scrubbing with a swab. The invaded cells were fixed and stained with 0.5 % crystal violet in 20 % methanol solution for 15 min, and counted per membrane.

### 2.5 RT-qPCR

Total RNA was isolated using NucleoSpin RNA (TaKaRa, Shiga, Japan). The RNA was treated with DNase I (TaKaRa), and cDNA was synthesized using random hexamer and oligo-dT primers and the ReverTra Ace qPCR RT Master Mix (TOYOBO, Osaka, Japan). Quantitative PCR (qPCR) was performed using a QuantStudio 5 Real-Time PCR system (Thermo Fisher Scientific) with the SYBR Green Real-Time PCR Master Mix -Plus- (TOYOBO). The comparative Ct method was used to determine the relative expression of *DRAIC* after normalization to *HPRT1*. The PCR primer sequences are as follows: *DRAIC* [8] forward 5′-TGAACTCAACTCCTGAGAAGGAC-3′ and reverse 5′-CGCTCTCAGACTCTTCAGTTCTC-3′; *HPRT1* [16] forward 5′-AGCCAGACTTTGTTGGATTTG-3′ and reverse 5′-TTTACTGGCGATGTCAATAGG-3′.

### 2.6 Gene expression microarray and Gene Ontology (GO) analysis

Total RNA from VMRC-LCD cells transfected with si-NC and si-*DRAIC*2 was isolated using NucleoSpin RNA. The RNA integrity was assessed by an Agilent Technologies 2100 Bioanalyzer (Agilent Technologies, Santa Clara, CA, USA), and gene expression analysis was performed using the Human Clariom S Assay (Affymetrix, Santa Clara, CA, USA). The raw data were obtained as CEL files, which were processed using the Signal Space Transformation Robust Multi-Chip Analysis (SST-RMA) algorithm in the Transcriptome Analysis Console software (Thermo Fisher Scientific) with background correction, quantile normalization, and summarization. The expression dataset was deposited into the National Center for Biotechnology Information Gene Expression Omnibus (NCBI GEO) (https://www.ncbi.nlm.nih.gov/geo/) and is accessible through GEO Series accession number GSE242691. GO analysis was performed using Metascape (https://metascape.org/gp/index.html#/main/step1) [26], with the upregulated (fold change >1.5) or downregulated (fold change < −1.5) genes in si-*DRAIC*2 compared with si-NC.

### 2.7 Chorioallantoic membrane (CAM) assay

The CAM assay was performed in accordance with the protocol that was approved by the Animal Affairs Committee at Fujita Health University (approval number APU22067). The fertilized eggs were obtained from the Fukushima branch of MORI BREEDING FARM (Fukushima, Japan). The CAM assay was performed as previously reported, but with modifications [27]. Briefly, the eggs were placed in the incubator (Rcom MX-20, BellBird, Japan) set at 37.5 °C, with 65 % humidity, and the eggs were rotated once an hour. On day 8 of incubation, egg rotation was stopped, and a round window was opened using the round bar to expose the CAM. PTEF O-ring (Sansho, Tokyo, Japan) was placed on the xenografted area of the CAM, and VMRC-LCD cells at 5.0 × 10^6^ in a total of 20 μL medium (10 μL PBS and 10 μL Matrigel basement matrix (Corning)) were xenografted. The shell window was covered with Tegaderm™ (3 M Japan Limited, Tokyo, Japan), and the egg was placed back into the incubator. On day 5 of the CAM assay, a tumor on the CAM was excised and the eggs were sacrificed in a −20 °C freezer for 2 h. The tumor was fixed using 10 % neutral buffered formalin for pathological analysis.

### 2.8 CISH

CISH was performed on the cell lines and TMA samples (FDA331, FDA808I-1, FDA808I-2, EN801b, and LC811a) (TissueArray.Com LLC, Derwood, MD, USA) with RNAscope (R) 2.5 HD Reagent Kit-BROWN (Advanced Cell Diagnostics (ACD)) (Newark, CA, USA). The Z probes for *DRAIC* (#568281) and *DapB* (negative control, #310043) were obtained from ACD. The Z probe for *DRAIC* that targeted 2–1270 of NR_026979.1 was designed and synthesized by ACD. The commonly available TMAs analyzed in this study have been widely used in research, and their academic value has already been determined. Therefore, the Institutional Review Board deemed that this study was not required for ethical review in light of the Ethical Guidelines for Medical and Biological Research Involving Human Subjects.

After deparaffinization, the samples were treated with H_2_O_2_ to block endogenous peroxidase activity. The target retrieval was performed for 3 min using the SR-MP300-K (Panasonic, Kadoma, Osaka, Japan). The samples were treated with the Protease Plus reagent for 15 min (cell lines) or 30 min (TMAs). Each Z probe was hybridized for 2 h at 40 °C in a HybEZ Hybridization System (ACD). The signal was amplified using AMP1 (30 min at 40 °C), AMP2 (15 min at 40 °C), AMP3 (30 min at 40 °C), AMP4 (15 min at 40 °C), AMP5 (60 min at RT), and AMP6 (15 min at RT) reagents. The signal was visualized using the 3,3′-diaminobenzidine (DAB) chromogen diluted in the DAB substrate buffer, and the slides were counterstained with hematoxylin.

Individually stained slides were scanned with Axioscan7 (Oberkochen, Germany) and visualized using QuPath open-source software [28]. *DRAIC* expression was analyzed by counting dark brown dots regardless of the dot size in both the nucleus and cytoplasm of 100 cells from 3 random fields of view and was indicated as the average [29]. Instances where the specimen detached during the CISH procedure were excluded from analysis. Instances with a large amount of hemosiderin and other biological pigments were also excluded from analysis because it was challenging to distinguish them from the *DRAIC* signal. Each slide was evaluated by two independent board-certified pathologists (K.S. and S·Y.).

## 3 Results

### 3.1 Expression of *DRAIC* in FFPE cell line block samples and xenograft tissues

**Fig. 1:**
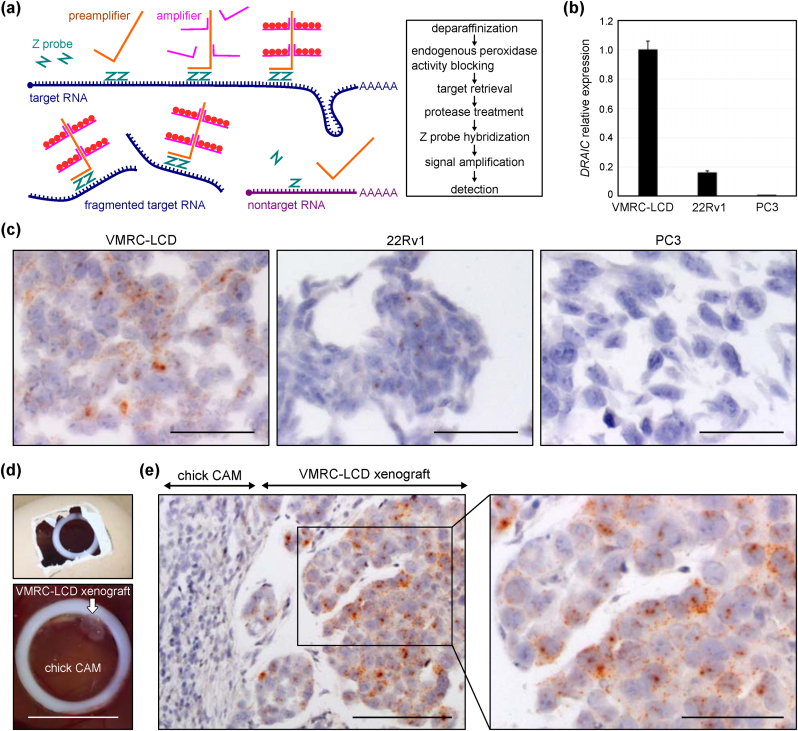
Expression of *DRAIC* in formalin-fixed paraffin-embedded (FFPE) cell line block and xenograft samples, (a) Schematic illustration of chromogenic *in situ* hybridization (CISH) using RNAscope. The RNAscope steps are shown in brief on the right. (b) The expression of *DRAIC* in VMRC-LCD, 22Rv1, and PC3 cell lines was measured by RT-qPCR and normalized to *HPRT1*. Mean ± standard deviation (S.D.) *n* = 3. The expression in VMRC-LCD was set as 1. (c) *DRAIC* staining of VMRC-LCD (left), 22Rv1 (center), and PC3 (right) was analyzed by CISH. Scale bar: 50 μm. (d) Chorioallantoic membrane (CAM) assay. Upper panel: the window was opened and a PTEF O-ring was placed on the CAM. Lower panel: the tumor mass (white arrow) of the VMRC-LCD xenograft on the chick CAM at day 5. Scale bar: 10 mm. (e) *DRAIC* staining in the CAM assay was analyzed by CISH. The region with a black line rectangle at the left panel is magnified right. Scale bar, 100 μm (left) and 50 μm (right).

We performed CISH using RNAscope, a technique to detect intracellular RNA with high sensitivity and specificity [24]. After two paired Z probes were tandemly hybridized to the target RNA, the L-shaped preamplifier binds to the upper region of the Z probes (Fig. 1a). The amplifiers then bind to multiple sites on each preamplifier, and the labeled probes containing a chromogenic enzyme bind to multiple sites on each amplifier, which makes it possible to detect low levels of RNA with high sensitivity. A pool of multiple paired Z probes can detect partially fragmented RNAs in an FFPE specimen. When the paired Z probes do not tandemly bind to RNA, the preamplifier does not bind, thus ensuring high specificity [24].

We first evaluated the expression of *DRAIC* in human cancer cell lines. Based on the *DRAIC* expression analyzed using the Cancer Cell Line Encyclopedia (CCLE) dataset [30], we selected three human cancer cell lines: VMRC-LCD, 22Rv1, and PC3 cells (Fig. S1). RT-qPCR showed that VMRC-LCD had the highest expression of *DRAIC*, 22Rv1 had moderate expression, and PC3 had the lowest expression (Fig. 1b). We performed the RNAscope CISH for *DRAIC* using the FFPE samples, which revealed that the strongest signal and largest number of dots were found in VMRC-LCD (Fig. 1c, left), weaker and fewer signals in 22Rv1 (Fig. 1c, center), and the weakest and least amount of signals in PC3 (Fig. 1c, right) (Fig. S2). As for the subcellular localization, diffuse small dots were predominantly observed in the cytoplasm, while some accumulated larger dots were detected in a nuclear compartment. As a negative control, we confirmed that no signal of *DapB*, a bacterial RNA, was observed in these three cell lines (Fig. S3).

We next detected *DRAIC* in the VMRC-LCD xenograft in the CAM assay. After xenografting VMRC-LCD cells onto the chick CAM, a macroscopically visible tumor formed at day 5 (Fig. 1d). CISH detected *DRAIC* signals in the VMRC-LCD xenograft but not in the chick CAM tissue (Fig. 1e). Similar subcellular localization patterns shown in Fig. 1c were observed, namely many diffuse small dots in the cytoplasm and larger dots in the nucleus.

### 3.2 Expression of *DRAIC* in TMAs

**Fig. 2:**
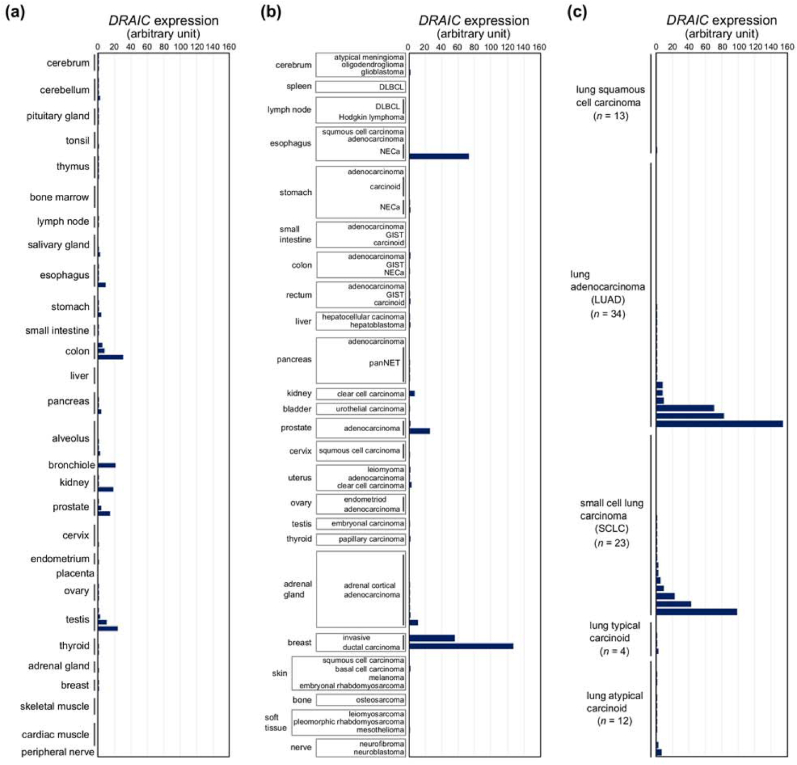
Expression of *DRAIC* in tissue microarrays (TMAs), (a)–(c) The expression of *DRAIC* was analyzed by CISH in normal tissues (a), various tumor tissues (excluding lung cancers) (b), and lung cancer tissues (c). Each bar indicates the average number of dots per 100 cells from three random fields of view. DLBCL; diffuse large B-cell lymphoma, NECa; neuroendocrine carcinoma, GIST; gastrointestinal stromal tumor, panNET; pancreatic neuroendocrine tumor.

**Fig. 3:**
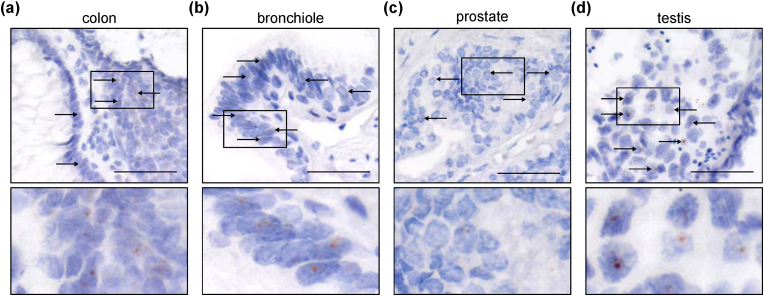
Representative images of *DRAIC* expression in normal tissues, Representative images of *DRAIC* expression in normal epithelial cells of the colon (a), bronchiole (b), prostate (c), and testis (d) tissues. Arrows indicate some of the *DRAIC* signals. Scale bar: 50 μm. The region with a black line rectangle is magnified below. The length of each magnified panel is 50 μm.

Based on the results using the FFPE cell block samples and xenograft tissues, we performed the RNAscope CISH on the TMAs to comprehensively evaluate the expression pattern of *DRAIC* in normal and tumor tissues. In Fig. 2 and Supplementary Tables, the results of normal tissues, various tumor tissues excluding lung cancer, and lung cancer tissues were summarized. In normal tissues (*n* = 89), *DRAIC* was weakly expressed in some epithelial cells of the colon, bronchiole, kidney, prostate, and testis and was mostly localized in the nucleus (Fig. 2, Fig. 3, and Table S1). *DRAIC* was not expressed in non-epithelial cells such as fibroblasts and blood cells.

**Fig. 4:**
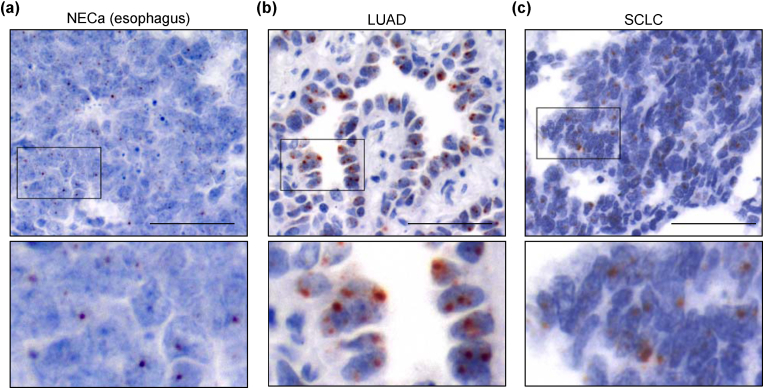
Representative images of *DRAIC* expression in cancerous tissues, Representative images of *DRAIC* expression in NECa of the esophagus (a), lung adenocarcinoma (LUAD) (b), and small cell lung carcinoma (SCLC) (c). Scale bar: 50 μm. The region with a black line rectangle is magnified below. The length of each magnified panel is 50 μm.

In tumor tissues (*n* = 155), *DRAIC* was moderately to highly expressed in some instances (Fig. 2b and c) (Tables S2 and S3). These included prostate adenocarcinoma (Fig. S4, left), invasive ductal carcinoma of the breast (Fig. S4, right), neuroendocrine carcinoma (NECa) of the esophagus (Fig. 4a), lung adenocarcinoma (LUAD) (Fig. 4b), and small cell lung carcinoma (SCLC) (Fig. 4c). The *DRAIC* signals were detected in both the cytoplasm and nucleus of these cancer cells, although the distribution patterns varied according to the sample.

### 3.3 Potential downstream genes of *DRAIC* in the neuroendocrine carcinoma-derived cell line, VMRC-LCD

**Fig. 5:**
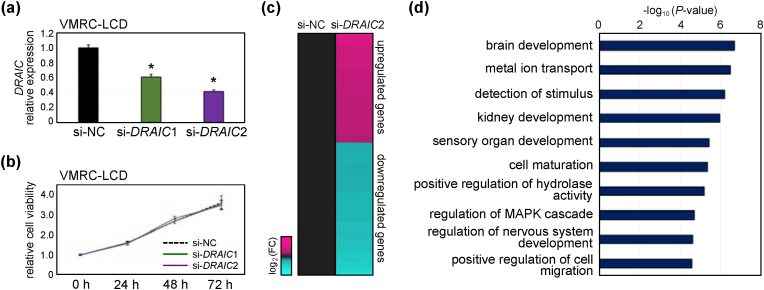
Effect of siRNA-mediated knockdown of *DRAIC* in a neuroendocrine-differentiated lung cancer cell line, VMRC-LCD, (a) The expression of *DRAIC* after transfection with siRNAs against *DRAIC* in VMRC-LCD cells was measured by RT-qPCR and normalized to *HPRT1.* Mean ± S.D. *n* = 3. The expression of the si-negative control (NC) was set as 1. *p < 0.05. (b) The cell viability of si-NC, si-*DRAIC*1, and si-*DRAIC*2 of the VMRC-LCD cells was measured using a WST-8 assay. The viability at 0 h of each siRNA-transfected cell was set as 1. (c) Heat map showing the upregulated (FC > 1.5) and downregulated (FC < −1.5) genes of si-*DRAIC*2 compared with si-NC in the VMRC-LCD cells. FC; fold change. (d) Gene Ontology terms for the Biological Processes enriched in the downregulated genes of si-*DRAIC*2.

Our recent findings based on *in silico* analysis revealed that a higher expression of *DRAIC* is associated with the neuroendocrine (NE) feature in lung cancer [31]. Because CISH detected the *DRAIC* signal in a subset of NECa and SCLC (NE-differentiated lung carcinoma) (Fig. 2, Fig. 4), we hypothesized that *DRAIC* may perform a biological role in NECa. Thus, we transfected siRNAs against *DRAIC* in VMRC-LCD, which is a NE-differentiated LUAD cell line [32]. Although two different siRNAs (si-*DRAIC*1 and si-*DRAIC*2) against *DRAIC* reduced *DRAIC* expression compared with the negative control (si-NC) (Fig. 5a), the cellular viability and invasive ability were not affected by *DRAIC* knockdown (Fig. 5b and Fig. S5). To investigate the potential downstream genes, we selected si-*DRAIC*2 for gene expression microarray because it showed the slightly greater effect on *DRAIC* knockdown than si-*DRAIC*1 (Fig. 5a). We found 1100 upregulated and 1343 downregulated genes in si-*DRAIC*2 compared with si–NC–treated VMRC-LCD cells (Fig. 5c). GO analysis showed the enrichment of GO terms such as brain development, regulation of nervous system development, cell maturation, MAPK, and cell migration in the downregulated genes (Fig. 5d). On the other hand, GO terms including regulation of hormone levels and regulation of cell-substrate adhesion were enriched in the upregulated genes (Fig. S6).

## 4 Discussion

Studies on the expression of *DRAIC* have been mainly conducted by RT-qPCR using total RNA from cell lines and clinical samples. Publicly available transcriptome data such as microarray and RNA-sequencing have also been used to analyze *DRAIC* expression [8,31]. Although these approaches are beneficial in understanding its overall expression tendency, detailed information regarding its expression patterns (e.g., cell type specificity) is not available. Furthermore, even in the worldwide databases, such as The Cancer Genome Atlas, not all benign and malignant histological types are collected.

In this study, we utilized RNAscope technology to detect *DRAIC* expression in FFPE specimens. RNAscope is an established technology to quantitatively analyze the expression of coding and non-coding RNA with high sensitivity and specificity [24,29,33,34]. Using this technology, we successfully detected *DRAIC* in FFPE cell block samples and xenograft tissues, and these expression levels were consistent with the CCLE dataset and our RT-qPCR results. VMRC-LCD, which had the highest expression levels of *DRAIC* in the analyzed cell lines, had many small dots in the cytoplasm. Since we showed in our cell fractionation assay that *DRAIC* was predominantly expressed in the cytoplasm of prostate cancer cells [8], several molecular mechanisms as a cytoplasmic lncRNA have been reported [9,10,14,15,35]. Importantly, this study revealed that *DRAIC* was also expressed in the nuclear compartment as larger dots. Although the precise region where the nuclear *DRAIC* was expressed has not been identified, this finding may lead to the understanding of novel functions of *DRAIC*, considering that nuclear lncRNAs regulate gene expression through multiple mechanisms [4,5]. Tiessen et al. demonstrated through cell fractionation analysis that *DRAIC* was expressed both in the nucleus and cytoplasm of a breast cancer cell line, MCF-7 [16]. These results indicate that the pattern of *DRAIC* subcellular localization might be cell-type dependent. While the molecular mechanisms of this unique pattern remain unclear, the sequence motif and its binding partners might affect the localization [36]. It would be important to analyze the intracellular distribution in each cell line by RNAscope CISH to determine the diversity of biological functions.

This is the first report analyzing the *DRAIC* expression on TMAs. In normal tissues, some epithelial cells weakly expressed *DRAIC,* mainly in their nucleus. In contrast, some specific cancer cells had moderate to high *DRAIC* expression in their nucleus and cytoplasm, although the distribution pattern varied. In addition to the adenocarcinomas of the prostate and breast, whose *DRAIC* expressions have been analyzed [8,13,37], this study demonstrated the high expression in a subset of NECa, LUAD, and SCLC. Our previous bioinformatics-based analysis indicated that *DRAIC* was highly expressed in ASCL1 (a NE-differentiation-associated transcription factor)-positive LUAD and SCLC [31]. Because RNAscope could be simultaneously performed with immunohistochemistry within the same specimen [38], it would be interesting to further demonstrate the positive correlation between *DRAIC* RNA and ASCL1 protein in these instances. In addition, by analyzing the infiltrating cell types, we plan to clarify whether *DRAIC* is an immune-related lncRNA because it was suggested to be involved in the immune cell infiltration in LUAD [17]. It would also be important to analyze *DRAIC* involvement in the prognosis of patients with LUAD because contradictory results have been presented in our [8,31] and other [15] reports.

We performed a gene expression microarray after *DRAIC* knockdown by siRNA in VMRC-LCD, an NE-differentiated LUAD cell line. The GO terms related to NE were enriched by knockdown, which suggests that *DRAIC* might be involved in the characteristics of cancer cells with NE-differentiation, although the molecular mechanisms are unknown. *DRAIC* knockdown also influenced the gene expression associated with cellular migration and cell-substrate adhesion, while cell viability was not affected. Consistent with these results, *DRAIC* knockout using the CRISPR/Cas9 system induced cell migration and anchorage-independent growth in prostate cancer cells, while cell proliferation at the standard plate was not altered [9]. Notably, *DRAIC* knockdown remained low invasive ability in VMRC-LCD cells, suggesting that the effect of *DRAIC* on cellular invasion is also cell type-dependent. Because *DRAIC* induces or represses cellular invasion, depending on cancer cell types [8,9,15,39], we should analyze the cellular phenotypes *in vitro* and *in vivo* using various NE-differentiated cancer cells. In addition, given that siRNA strategies are not effective in knocking down nuclear RNAs because RNAi-machinery is localized in the cytoplasm [40,41], we need to knockout *DRAIC* using CRISPR/Cas9 in VMRC-LCD and other cell lines to fully analyze the functions of nuclear *DRAIC*. Nevertheless, our gene expression analysis presented here suggests the potential involvement of *DRAIC* in NE-differentiation and cancer-related phenotypes.

## 5 Conclusion

We successfully detected *DRAIC* expression by RNAScope CISH in FFPE cell line samples and TMAs. Because this study aimed to establish RNAscope CISH for *DRAIC* and investigate expression patterns in various cancers, the case numbers of each histological type were limited. Whether and how *DRAIC* expression is changed during carcinogenesis and involved in the prognosis of patients will be investigated next using a larger number of FFPE specimens. Although further analyses are required to fully understand *DRAIC* expression and function, our results provide novel insights into the molecular mechanisms of *DRAIC* in physiological and disease conditions.

## Informed consent statement

Tissue microarrays commercially obtained from TissueArray.Com LLC were developed in compliance with their ethical standards with the donor's informed consent and privacy.

## Funding

This research was funded by Grants-in-Aid for Scientific Research from the 10.13039/501100001691Japan Society for the Promotion of Science (grant number 15H06717 and 21K09862).

## CRediT authorship contribution statement

**Kouhei Sakurai:** Conceptualization, Formal analysis, Funding acquisition, Investigation, Methodology, Resources, Validation, Visualization, Writing – original draft. **Seiji Yamada:** Conceptualization, Formal analysis, Investigation, Methodology, Validation, Writing – review & editing. **Rika Ito:** Formal analysis, Investigation, Validation, Visualization. **Mako Ochiai:** Formal analysis, Investigation, Validation, Visualization. **Tatsuya Ando:** Methodology, Writing – review & editing. **Yasuhiro Sakai:** Methodology, Writing – review & editing. **Taku Kato:** Methodology, Resources, Writing – review & editing. **Hiroyasu Ito:** Methodology, Writing – review & editing.

## Declaration of competing interest

The authors declare that they have no known competing financial interests or personal relationships that could have appeared to influence the work reported in this paper.
