# Supplementary material for: Chromogenic in situ hybridization reveals specific expression pattern of long non-coding RNA DRAIC in formalin-fixed paraffin-embedded specimen
Source: Noncoding RNA Res. 2023 Nov 11;9(1):76–83. doi: 10.1016/j.ncrna.2023.11.004 (PMC10700117; doi:10.1016/j.ncrna.2023.11.004)
Supplement: Multimedia component 1 [file mmc1.pdf]

Supplementary material

Chromogenic *in situ* hybridization reveals specific expression pattern of long non-coding RNA *DRAIC* in formalin-fixed paraffin embedded specimen

Kouhei Sakurai, Seiji Yamada, Rika Ito, Mako Ochiai, Tatsuya Ando, Yasuhiro Sakai, Taku Kato, and Hiroyasu Ito

Corresponding authors
Kouhei Sakurai
Department of Joint Research Laboratory of Clinical Medicine, School of Medicine,
Fujita Health University
470-1192, 1-98 Dengakugakubo, Kutsukake-cho, Toyoake, Aichi, Japan.
Tel: +81562-93-9934
e-mail: kouhei.sakurai@fujita-hu.ac.jp

Seiji Yamada
Department of Diagnostic Pathology, School of Medicine,
Fujita Health University
470-1192, 1-98 Dengakugakubo, Kutsukake-cho, Toyoake, Aichi, Japan.
Tel: +81 562-93-2319
e-mail: yamadas@fujita-hu.ac.jp

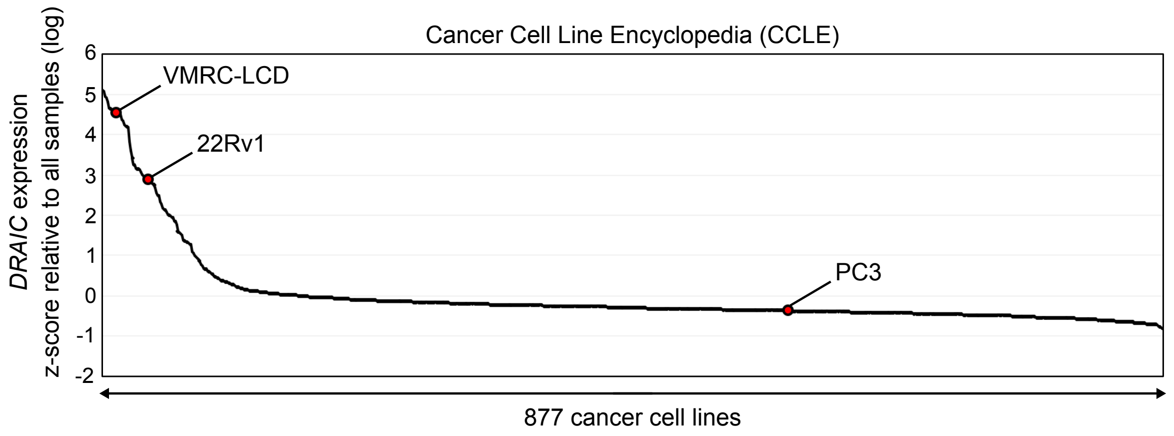

Figure S1

***DRAIC* expression across 877 human cancer cell lines from the Cancer Cell Line Encyclopedia (CCLE) dataset**

DRAIC expression was measured using the CCLE microarray dataset (Novartis/Broad, Nature 2012) and visualized using cBioPortal (https://www.cbioportal.org/).

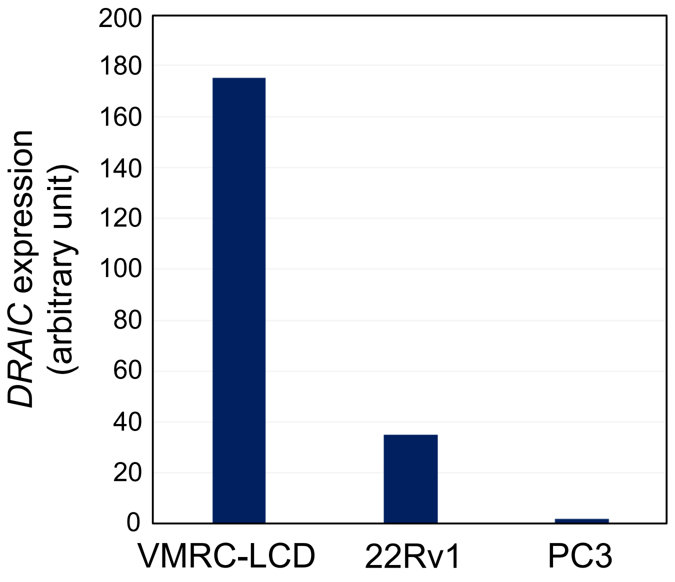

Figure S2

Expression of *DRAIC* in cell line block samples

The expression of *DRAIC* in VMRC-LCD, 22Rv1, and PC3 cell line block samples was analyzed by counting dark brown dots regardless of the dot size in both the nucleus and cytoplasm of 100 cells from 3 random fields of view and indicated as the average.

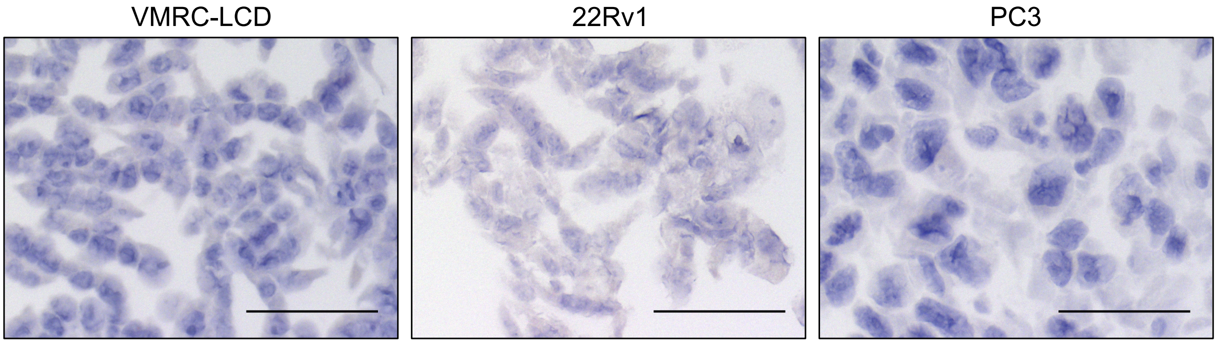

Figure S3

RNAscope CISH for *DapB* in the VMRC-LCD, 22Rv1, and PC3 cell lines

DapB, a bacterial RNA, was not detected in the FFPE cell block samples, VMRC-LCD, 22Rv1, and PC3. Scale bar: 50 μm.

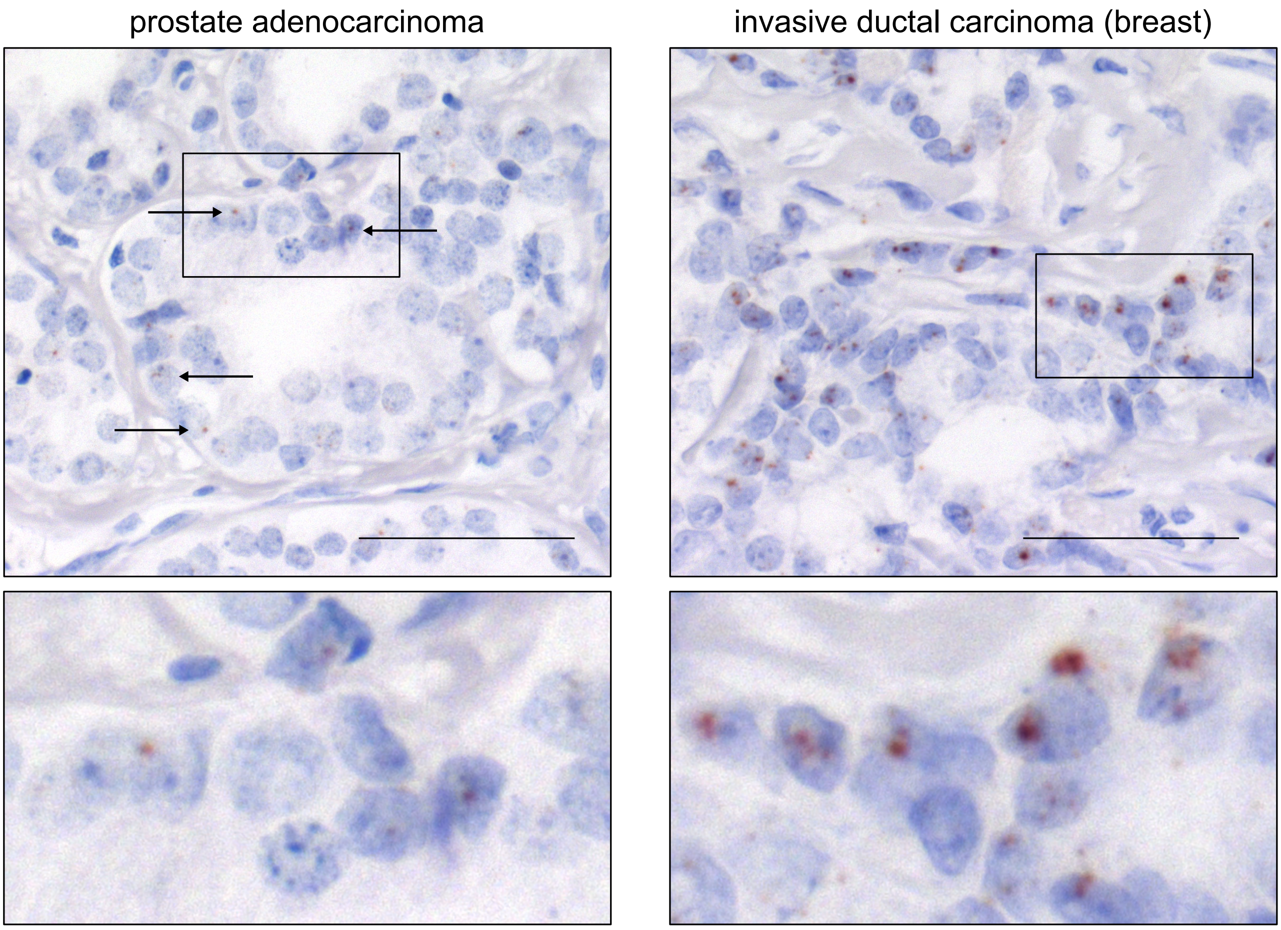

Figure S4

***DRAIC* expression in adenocarcinomas of the prostate and breast**

Representative images of *DRAIC* expression in prostate adenocarcinoma (left) and invasive ductal carcinoma of the breast (right). Arrows indicate some of the *DRAIC* signals in the prostate adenocarcinoma. Scale bar: 50 μm. The region with a black line rectangle is magnified below. The length of each magnified panel is 50 μm.

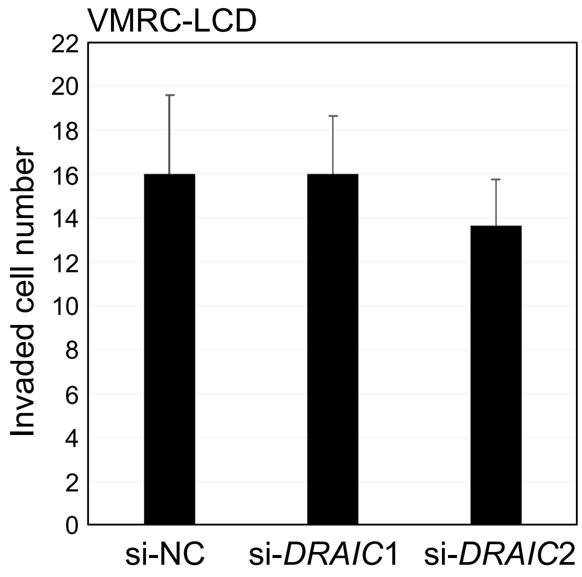

Figure S5

Matrigel invasion assay with VMRC-LCD-siNC, -si-*DRAIC*1, and -si-*DRAIC*2 cells

Knockdown of *DRAIC* did not affect invasive ability in VMRC-LCD cells. Mean ± S.D. *n* = 3.

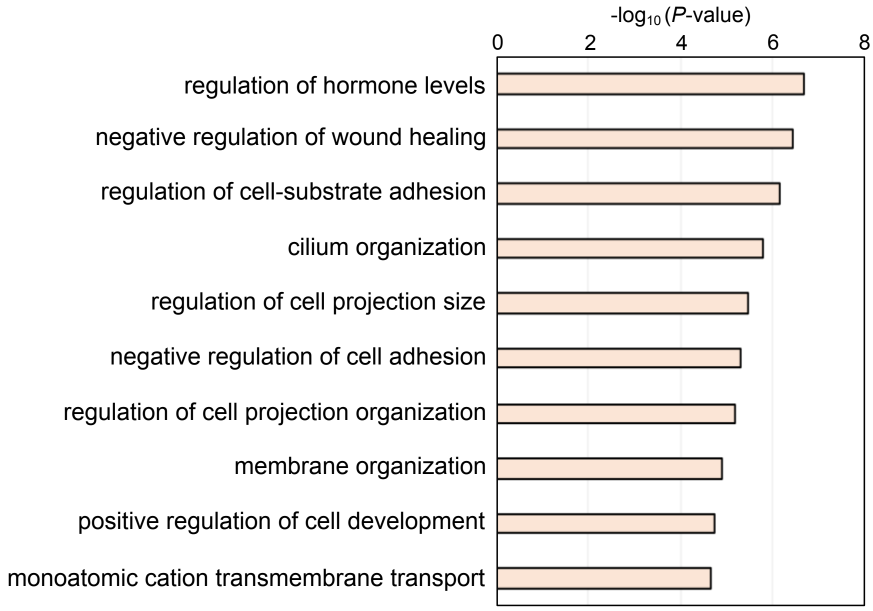

Figure S6

Gene Ontology (GO) analysis of siRNA-mediated knockdown of *DRAIC* in VMRC-LCD cells

GO terms for Biological Processes enriched in the upregulated genes in si-*DRAIC*2 were analyzed and visualized using Metascape (https://metascape.org/gp/index.html#/main/step1).

Table S1

DRAIC expression in normal tissues

organ	age	sex	*DRAIC* score
cerebrum	50	F	0.33
cerebrum	38	F	0.67
cerebrum	49	M	1.67
cerebellum	32	M	0.33
cerebellum	56	F	0.67
cerebellum	24	F	0.67
cerebellum	45	M	3.33
pituitary gland	25	F	0.33
pituitary gland	21	F	0.33
pituitary gland	40	F	0.33
tonsil	21	F	0
tonsil	45	M	0
tonsil	7	F	2
thymus	15	F	0.33
thymus	16	M	1
thymus	10m	M	1
thymus	45	M	1
bone marrow	21	F	0
bone marrow	61	F	0
bone marrow	70	M	0
bone marrow	15	M	0
lymph node	35	M	0.33
lymph node	25	F	1.33
salivary gland	35	M	0
salivary gland	23	M	0
salivary gland	40	M	1.33
salivary gland	54	F	2.33
esophagus	30	M	0.33
esophagus	24	M	0.33
esophagus	34	M	0.67
esophagus	39	F	9.67
stomach	37	F	0
stomach	24	M	0.33
stomach	38	M	2
stomach	30	M	4.67
small intestine	45	M	1
small intestine	21	F	1
colon	35	M	5.33
colon	45	M	7.67
colon	55	M	31
liver	43	M	0
liver	35	M	0
liver	40	M	0
pancreas	27	M	0
pancreas	21	F	1.67
pancreas	40	F	2
pancreas	49	F	4.33
alveolus	40	F	0
alveolus	33	M	0
alveolus	21	F	0
alveolus	21	F	0.33
alveolus	21	F	1
alveolus	48	M	3

organ	age	sex	*DRAIC* score
bronchiole	21	F	21.67
kidney	47	M	1
kidney	50	M	1
kidney	38	M	19.33
prostate	43	M	0.33
prostate	35	M	3.67
prostate	31	M	15.33
cervix	36	F	0
cervix	72	F	0
cervix	37	F	0
cervix	65	F	0.33
endometrium	48	F	0
endometrium	41	F	0.67
placenta	4m	M	0
ovary	35	F	0.67
ovary	64	F	0.67
ovary	45	F	1.67
testis	35	M	0.33
testis	74	M	2.67
testis	65	M	11
testis	47	M	24.33
thyroid	22	M	0
thyroid	40	F	1
thyroid	35	M	1
adrenal gland	73	M	0
adrenal gland	43	M	0.33
breast	35	F	1.33
breast	29	F	2
skeletal muscle	40	M	0
skeletal muscle	49	F	0
skeletal muscle	50	M	0
cardiac muscle	35	F	0
cardiac muscle	26	M	0
cardiac muscle	40	M	0
cardiac muscle	47	M	0
peripheral nerve	42	F	0

Table S2

DRAIC expression in various tumor tissues (excluding lung cancers)

organ	age	sex	pathological diagnosis	TNM	stage	*DRAIC* score
cerebrum	65	F	atypical meningioma	-	-	0
cerebrum	39	F	oligodendroglioma	-	-	0
cerebrum	17	M	glioblastoma	-	-	1.67
spleen	21	M	diffuse large B-cell lymphoma	-	-	0
lymph node	55	M	diffuse large B-cell lymphoma	-	-	0
lymph node	50	F	diffuse large B-cell lymphoma	-	-	0
lymph node	27	M	Hodgkin lymphoma	-	-	0
esophagus	58	M	squamous cell carcinoma	T3N0M0	IIA	0
esophagus	63	M	adenocarcinoma	T3N0M0	IIB	0
esophagus	53	F	neuroendocrine carcinoma	T2N0M0	IB	0
esophagus	55	M	neuroendocrine carcinoma	T1N0M0	IB	73.67
stomach	73	F	mucinous adenocarcinoma	T2N1M0	IIA	0
stomach	50	M	carcinoid	T4N0M0	IIB	0
stomach	64	F	carcinoid	T2N0M0	I	0
stomach	52	F	carcinoid	T2N0M0	I	0
stomach	68	M	neuroendocrine carcinoma	T3N1M0	IIIB	0.33
stomach	68	M	neuroendocrine carcinoma	T3N1M0	IIIB	2
small intestine	64	F	adenocarcinoma	T4N0M0	II	0
small intestine	71	F	gastrointestinal stromal tumors, GIST	T2N0M0	IIB G2	0
small intestine	57	M	carcinoid	T2N0M0	I	0
colon	43	M	adenocarcinoma	T3N1M0	IIIB	1.33
colon	54	M	gastrointestinal stromal tumors, GIST	T2N0M0	IIB G2	0
colon	63	F	neuroendocrine carcinoma	T3N0M0	IIA	1
rectum	36	M	adenocarcinoma	T2N0M0	I	0
rectum	63	F	gastrointestinal stromal tumors, GIST	T2N0M0	IIB G2	0.67
rectum	70	F	carcinoid	T3N0M0	IIA	2
liver	55	F	hepatocellular carcinoma	T3aN0M0	IIIA	0.33
liver	17	F	hepatoblastoma	-	-	1.33
pancreas	64	M	ductal adenocarcinoma	T3N0M0	IIA	0
pancreas	52	F	pancreatic neuroendocrine tumor	-	-	1
pancreas	30	M	pancreatic neuroendocrine tumor	-	-	0.67
pancreas	40	F	pancreatic neuroendocrine tumor	-	-	0.33
pancreas	15	F	pancreatic neuroendocrine tumor	-	-	0
pancreas	16	F	pancreatic neuroendocrine tumor	-	-	0
kidney	60	M	clear cell carcinoma	T2N0M0	II	6.67
bladder	58	F	invasive low grade urothelial carcinoma	T1N0M0	I	0.33
prostate	80	M	adenocarcinoma (Gleason grade:4）	T2N1M1c	IVB	1.67
prostate	77	M	adenocarcinoma (Gleason grade:3）	-	-	26
cervix	36	F	squamous cell carcinoma	-	-	0
cervix	49	F	squamous cell carcinoma	T2N0M0	II	1
uterus	56	F	leiomyoma	T2bN0M0	III	1.67
uterus	50	F	adenocarcinoma	T1bN0M0	IB	0.67
uterus	57	F	clear cell carcinoma	T3aN0M0	IIIA	3.33
ovary	62	F	endometrioid adenocarcinoma	T1aN0M0	IA	0
ovary	29	F	endometrioid adenocarcinoma	T3N0M0	III	0
testis	30	M	embryonal carcinoma	T2N0M0	I	0.67
thyroid	46	F	papillary carcinoma	T1bN0M0	I	2
adrenal gland	45	M	adrenal cortical adenocarcinoma	-	-	0
adrenal gland	45	M	adrenal cortical adenocarcinoma	-	-	0
adrenal gland	35	M	adrenal cortical adenocarcinoma	-	-	0
adrenal gland	59	M	adrenal cortical adenocarcinoma	-	-	0
adrenal gland	48	F	adrenal cortical adenocarcinoma	-	-	0.33
adrenal gland	54	M	adrenal cortical adenocarcinoma	-	-	0.67

adrenal gland	58	M	adrenal cortical adenocarcinoma	-	-	0.67
adrenal gland	44	M	adrenal cortical adenocarcinoma	-	-	0.67
adrenal gland	10	F	adrenal cortical adenocarcinoma	-	-	1.33
adrenal gland	53	F	adrenal cortical adenocarcinoma	-	-	11
breast	42	F	invasive ductal carcinoma	T2N1M0	IIB	56
breast	58	F	invasive ducta carcinoma	T2N1M0	IIB	127
skin	64	M	squamous cell carcinoma	T3N0M0	II	0
skin	85	F	basal cell carcinoma	T2N0M0	II	1.33
skin	70	F	malignant melanoma	T4N0M0	II	0
skin	20	F	embryonal rhabdomyosarcoma	T1bN0M0	IIA G3	0
bone	37	F	osteosarcoma	T2N0M0	IB	0
soft tissue	60	F	leiomyosarcoma	T2bN0M0	IIB G2	0
soft tissue	48	F	pleomorphic rhabdomyosarcoma	T2N0M0	IIB G3	0
soft tissue	60	M	malignant mesothelioma	T2N0M0	II	0.33
nerve	21	M	neurofibroma	-	-	0
nerve	1	F	neuroblastoma	T3N0M0	-	0

- not available

Table S3

DRAIC expression in lung cancer tissues

organ	age	sex	pathological diagnosis	TNM	Stage	*DRAIC* score
lung	68	M	squamous cell carcinoma	T2N0M0	IB	0
lung	68	M	squamous cell carcinoma	T2N0M0	IB	0
lung	67	M	squamous cell carcinoma	T3N0M0	IIB	0
lung	74	M	squamous cell carcinoma	T2N0M0	IB	0
lung	56	M	squamous cell carcinoma	T3N2M0	IIIA	0
lung	65	M	squamous cell carcinoma	T3N0M0	IIB	0
lung	69	M	squamous cell carcinoma	T1N0M0	IA	0
lung	51	F	squamous cell carcinoma	T2N1M0	IIA	0
lung	48	M	squamous cell carcinoma	T2N0M0	IB	0
lung	62	M	squamous cell carcinoma	T2N1M0	IIA	0
lung	66	M	squamous cell carcinoma	T3N1M0	IIIA	0
lung	54	M	squamous cell carcinoma	T3N0M0	IIB	0
lung	64	M	squamous cell carcinoma	T2bN0M0	IIA	0.33
lung	71	F	adenocarcinoma	T2N1M0	IIA	0
lung	35	F	adenocarcinoma	T2N1M0	IIA	0
lung	59	M	adenocarcinoma	T4N3M0	IIIB	0
lung	43	F	adenocarcinoma	T2N2M0	IIIA	0
lung	58	M	adenocarcinoma	T1N0M0	IA	0
lung	49	F	adenocarcinoma	T2N1M0	IIA	0
lung	75	M	adenocarcinoma	T2N0M0	IB	0
lung	65	M	adenocarcinoma	T2N2M0	IIIA	0
lung	67	M	adenocarcinoma	T2N0M0	IB	0
lung	57	M	adenocarcinoma	T2N0M0	IB	0
lung	82	M	adenocarcinoma	T4N1M0	IIIA	0
lung	47	M	invasive adenocarcinoma	T2N0M0	IB	0
lung	42	M	invasive adenocarcinoma	T2aN0M0	IB	0
lung	56	F	papillary adenocarcinoma	T2N0M0	IB	0
lung	56	F	papillary adenocarcinoma	T2N1M0	IIA	0
lung	67	M	lepidic predominant invasive adenocarcinoma	T2N0M0	IB	0
lung	48	F	lepidic predominant invasive adenocarcinoma	T2N0M0	IB	0
lung	67	M	lepidic predominant invasive adenocarcinoma	T2N1M0	IIA	0
lung	65	F	adenocarcinoma	T2N2M0	IIIA	0.33
lung	56	F	adenocarcinoma	T1bN0M0	IA	0.33
lung	77	M	adenocarcinoma	T3N0M0	IIB	0.33
lung	53	M	adenocarcinoma	T2N0M0	IB	0.33
lung	50	F	lepidic predominant invasive adenocarcinoma	T2N0M0	IB	0.33
lung	55	M	adenocarcinoma	T2N0M0	IB	0.67
lung	52	F	adenocarcinoma	T3N1M0	IIIA	0.67
lung	57	F	papillary adenocarcinoma	T2N0M0	IB	1
lung	54	F	adenocarcinoma	T2N1M0	IIA	1.67
lung	62	M	adenocarcinoma	T2aN0M0	IB	1.67
lung	64	F	adenocarcinoma	T2N1M0	IIA	8
lung	50	F	adenocarcinoma	T2N0M0	IB	8
lung	70	M	adenocarcinoma	T1N0M0	IA	9
lung	43	M	adenocarcinoma	T2aN0M0	IB	71.33
lung	54	M	invasive adenocarcinoma	T3N1M0	IIIA	83
lung	75	F	lepidic predominant invasive adenocarcinoma	T2N1M0	IIA	154.33
lung	52	F	small cell carcinoma	T2N1M0	IIA	0
lung	51	M	small cell carcinoma	T4N1M0	IIIA	0
lung	37	F	small cell carcinoma	T2N0M0	IB	0
lung	53	F	small cell carcinoma	T3N2M0	IIIA	0
lung	33	M	small cell carcinoma	T2N2M0	IIIA	0
lung	55	M	small cell carcinoma	T2N1M0	IIA	0
lung	49	M	small cell carcinoma	T2N0M0	IB	0

lung	60	M	small cell carcinoma	T2N0M0	IB	0
lung	61	M	small cell carcinoma	T4N0M0	IIIA	0
lung	64	M	small cell carcinoma	T3N0M0	IIB	0
lung	44	F	small cell carcinoma	T1N0N0	IA	0.33
lung	64	M	small cell carcinoma	T3N1M0	IIIA	0.33
lung	47	F	small cell carcinoma	T2N0M0	IB	0.33
lung	36	M	small cell carcinoma	T2N1M0	IIA	0.33
lung	60	M	small cell carcinoma	T3N1M0	IIIA	1.67
lung	53	M	small cell carcinoma	T3N0M0	IIB	2
lung	47	M	small cell carcinoma	T4N1M0	IIIA	2.33
lung	51	F	small cell carcinoma	T2N1M0	IIA	2.67
lung	43	F	small cell carcinoma	T2N1M0	IIA	5.33
lung	36	M	small cell carcinoma	T2N2M0	IIIA	9.67
lung	31	F	small cell carcinoma	T2N0M0	IB	23
lung	41	M	small cell carcinoma	T4N2M0	IIIB	43
lung	61	M	small cell carcinoma	T2N0M0	IB	99
lung	39	M	typical carcinoid	T2N0M0	IB	0
lung	48	F	typical carcinoid	T2N0M0	IB	0.33
lung	52	F	typical carcinoid	T2N0M0	IB	0.67
lung	71	M	typical carcinoid	T2N0M0	IB	3
lung	53	M	atypical carcinoid	T2N0M0	IB	0
lung	67	M	atypical carcinoid	T2N0M0	IB	0
lung	75	F	atypical carcinoid	T2N0M0	IB	0
lung	57	M	atypical carcinoid	T1N0M0	IB	0.33
lung	60	M	atypical carcinoid	T2N0M0	IB	0.33
lung	53	M	atypical carcinoid	T2N0M0	IB	0.33
lung	58	M	atypical carcinoid	T2N1M0	IIA	0.33
lung	64	M	atypical carcinoid	T2N0M0	IB	0.33
lung	72	M	atypical carcinoid	T2N0M0	IB	0.33
lung	60	M	atypical carcinoid	T2N0M0	IB	1
lung	59	M	atypical carcinoid	T2N0M0	IB	2.67
lung	42	M	atypical carcinoid	T2N1M0	IIA	7
